# The potential usefulness of standardized assessments to measure participant outcomes of adaptive/therapeutic horseback riding: a survey study

**DOI:** 10.3389/fvets.2023.1303991

**Published:** 2023-11-30

**Authors:** Moriah R. Hanson, Kathy Alm, Beth Fields, Robin Gabriels, Arlene A. Schmid, Lorann Stallones, B. Caitlin Peters

**Affiliations:** ^1^Department of Occupational Therapy, Colorado State University, Fort Collins, CO, United States; ^2^Professional Association of Therapeutic Horsemanship International, Denver, CO, United States; ^3^Department of Kinesiology, University of Wisconsin-Madison, Madison, WI, United States; ^4^Anschutz Medical Campus, University of Colorado School of Medicine, Aurora, CO, United States; ^5^Department of Psychology, One Health Institute, Colorado State University, Fort Collins, CO, United States; ^6^Temple Grandin Equine Center, Department of Animal Sciences, Colorado State University, Fort Collins, CO, United States

**Keywords:** therapeutic riding, adaptive riding, equine-assisted services, adaptive recreation, standardized assessment

## Abstract

Adaptive or therapeutic riding (A/TR) is a recreational activity which provides mounted and ground-based horsemanship opportunities adapted to the abilities of the participants. A/TR provides physical and psychological benefits to participants with diverse disabilities, including physical, developmental, cognitive, and age-related disabilities, promoting higher quality of life. A/TR professionals may be limited in their capacity to implement outcome assessments and report the benefits of their community-based A/TR services to a broad audience. The purpose of this study was to identify whether and how A/TR professionals currently measure participant outcomes; benefits and barriers to implementing standardized assessments in A/TR; and characteristics which would make assessments useful in the community-based A/TR environment. To address this purpose, we conducted a survey among A/TR professionals. We found that while A/TR professionals measure outcomes among their participants, they typically do not use standardized assessments. Survey respondents believed benefits of implementing standardized assessments included bolstering the A/TR profession, acquiring funding, and communicating about A/TR services to a broad audience. Respondents also identified several barriers to implementing standardized assessments including time, systemic, and expertise constraints. Respondents reported that useful standardized assessments would be relevant to all age groups and populations who receive A/TR services. Finally, respondents shared that for standardized assessments to be useful, they would need to be low-cost, require less than 10–20 min, and available in either paper or computer format. This study revealed that standardized assessments may be a strong support to the A/TR profession; however, assessments must meet the unique needs of A/TR professionals.

## 1 Introduction

Adaptive riding, or therapeutic riding (A/TR), is a recreational activity in which horseback riding is adapted to the needs of participants with diverse abilities and diagnoses (1). A/TR typically occurs in a group setting, with a therapeutic riding instructor teaching skills that address horsemanship goals, allowing diverse populations to access the natural benefits of horsemanship (1). A/TR provides an engaging recreational activity for people with diverse disabilities, such as individuals with autism spectrum disorder or cerebral palsy, veterans with post-traumatic stress disorder, and older adults with dementia (2). Furthermore, A/TR has been proposed to improve a variety of outcomes, including: self-confidence, motivation, courage, social involvement, self-perceived physical competency, and gross motor function (3–6). There are different levels of certifications available to therapeutic riding instructors; certified therapeutic riding instructors (CTRIs) are certified for entry-level riding instruction, while advanced (ATRIs) and master (MTRIs) instructors can provide intermediate/advanced instruction and program management, and MTRIs have even further expertise in equine-assisted services broadly.

Many researchers have identified broad benefits of participating in A/TR (3–6); however, therapeutic riding instructors and A/TR centers are limited in their capacity to gather and report the benefits of their community-based services. The field of human-animal interaction research has often identified the need for increased use of valid and reliable outcomes assessments that are used consistently across studies, in order to advance the field (7). This same need for valid, reliable, and consistent outcomes measures exists in community-based practice, but the needs of community-based professionals differ from the needs of researchers. The assessments often used in A/TR research may not be useful in a community-based A/TR setting. For example, many assessments used in research require administrators to have advanced training in standardized assessments (8), which is not required to become a CTRI/ATRI/MTRI. Additionally, some assessments used in A/TR research are validated for a specific age range or diagnosis, whereas A/TR centers often serve many different populations concurrently. Finally, many of the assessments used in research are costly and may not be sustainable for long-term use in the A/TR context. Due to these limitations, A/TR professionals currently do not have standardized assessments that can be feasibly used in their community-based context. Therefore, there is a need to identify high-quality standardized assessments which A/TR professionals can use to measure participant outcomes of their services; identifying such assessments could bolster the profession’s credibility and facilitate improved communication about the benefit of A/TR services to participants, funders, and the community.

A key consideration when identifying standardized assessments to be used in community-based services is the *clinical usefulness* of the assessment (9). Clinical usefulness is defined as whether an assessment improves the quality of services, is acceptable to administrators and participants, can improve the quality of services, and is worth the cost of its use. The purpose of the current study was to gather feedback from A/TR professionals pertaining to elements of standardized assessments which make them *clinically useful* to measure participant outcomes of community-based A/TR. To achieve this aim, we asked the following research questions:

1. How are CTRIs/ATRIs/MTRIs currently measuring participant outcomes, if at all?
2. Do A/TR interested parties believe it is important to identify standardized assessments to measure participant outcomes of A/TR? If so, what participant outcomes do A/TR interested parties believe are most important to assess and most likely to change as a result of A/TR participation?
3. What do A/TR interested parties believe would be benefits of, and barriers to, implementing standardized assessments to measure participant outcomes of TR?
4. What qualities of standardized assessments would be most useful in the community-based A/TR setting (e.g., for what populations is the assessment validated, frequency of assessment, assessment length and cost, etc.)?

## 2 Methods

### 2.1 Study design and participants

The Temple Grandin Equine Center (TGEC) at Colorado State University (CSU) and the Professional Association of Therapeutic Horsemanship, International (PATH, Intl.) formed a working group of A/TR interested parties. The working group was developed and met prior to this study. The ongoing work group consists of 11 members, selected because they represent different perspectives related to A/TR, including CTRIs, leadership from PATH, Intl. centers, researchers, and representatives from: TGEC, PATH Intl, the American Hippotherapy Association Inc., and the Horses and Humans Research Foundation. This work group provided feedback that greatly influenced the methods presented below.

This study was approved by the CSU Institutional Review Board (#3229) and a study survey was distributed to A/TR interested parties. To be eligible to complete the survey, respondents were required to be age 18 or older, understand English, and self-identify as one of the following A/TR interested parties: CTRI, center leadership, A/TR volunteer, A/TR participant, or caregiver of an A/TR participant. To be included in analyses, survey respondents had to complete at least 50% of the survey. Survey responses were submitted anonymously.

### 2.2 Data collection

The survey was created in Research Electronic Data Capture (REDCap), a secure, web-based software platform (10, 11). The survey was distributed to a national mailing list of A/TR interested parties maintained by PATH, Intl., the educational and credentialing body for A/TR professionals and centers. The email invitation included details of the study, including that participation was voluntary and 1 respondent would be randomly selected to receive a $100 gift card; clicking the survey link indicated consent. The survey was open for 8 weeks. Survey questions fell into five categories: (1) general information about the respondent; (2) if and how the respondent currently measures A/TR outcomes (for CTRIs and PATH., Intl leadership only); (3) the perceived importance and likelihood to change of possible participant outcome constructs (e.g., horsemanship skills, social skills, physical improvements); (4) benefits of and barriers to implementing standardized outcome assessments in community-based A/TR; and (5) considerations that may affect the usefulness of standardized assessments in the A/TR context (e.g., time and cost, etc.). The survey consisted of Likert-scale questions, “Select all that apply” questions, “Yes or No” questions, and open-ended short-answer questions (e.g., “Please describe,” or “Please list…”). The survey included 107 distinct questions, and operated using branching logic, such that each respondent answered a set of questions based on their previous answers; for example, respondents who self-identified as a CTRI were presented with different questions than those who self-identified as an A/TR participant. Depending on their answers, respondents were presented with an average of 50 questions. The survey allowed respondents to save their responses and return at a later time.

### 2.3 Data analysis

Survey data were exported from REDCap and downloaded to Microsoft Excel for analysis. We calculated descriptive statistics such as frequencies, percentages, medians, and interquartile ranges (IQR). For “Select all that apply” questions, total percentages across all response options summed greater than 100%, as respondents were allowed to select multiple responses. For short-answer questions, we category-coded responses into pre-existing answer options or created new codes derived from participant answers; we report category counts to summarize the short-answer responses (12).

## 3 Results

### 3.1 Respondent characteristics

Three hundred forty-seven total respondents completed portions of the survey. Two hundred seventy-seven participants completed at least 50% of the survey and therefore, were included in further analyses. Two hundred sixteen (78%) respondents identified as A/TR instructors, 99 (36%) identified as PATH, Intl. Center staff, 23 (8%) identified as A/TR volunteers, and 8 (3%) identified as either an A/TR participant or a caregiver of an A/TR participant. Due to the dearth of volunteer, participant, and caregiver respondents, we chose to focus all further analyses on respondents who identified as either A/TR instructors or PATH, Intl. center staff. A total of 233 A/TR instructors or PATH, Intl. center staff completed at least 50% of the survey, and 221 completed the entire survey. The remainder of the manuscript provides the results from these 233 A/TR instructors or PATH, Intl Center leadership.

Of the 216 respondents who identified as A/TR instructors, 200 (93%) identified as CTRIs; 27 (13%) identified as Advanced Therapeutic Riding Instructors (ATRIs), and 11 (5%) identified as Master Therapeutic Riding Instructors (MTRIs). These instructor types were combined for further analysis and will be hereafter identified as CTRIs. One hundred forty-one (65%) respondents reported being employed at a PATH, Intl. A/TR center, 37 (17%) reported working at a non-PATH, Intl. A/TR center, 22 (10%) reported contracting with PATH, Intl. centers, and 22 (10%) reported not currently working with an A/TR center.

### 3.2 Current assessments

One hundred and ninety-one respondents (82%) reported they currently measure participant outcomes of A/TR. Table 1 includes participant outcome constructs currently assessed by survey respondents.

**Table 1:** A/TR outcome constructs currently assessed by A/TR professionals.

Outcome construct	Number of responses (%) N = 191
Horsemanship skills	178 (93%)
Cognitive skills	156 (82%)
Communication skills	151 (79%)
Physical outcomes	142 (74%)
Emotional regulation	125 (65%)
Social outcomes	122 (64%)
Self-efficacy outcomes	110 (58%)
Recreation/leisure outcomes	105 (55%)
Empathy outcomes	89 (47%)
Quality of life outcomes	84 (44%)
Activities of daily living outcomes	45 (24%)
Community integration	38 (20%)
Instrumental activities of daily living outcomes	27 (14%)
Other	7 (4%)

“Other” outcomes included transference of skills to daily life, independence, and mental health.

Among the 191 survey respondents who reported tracking participant outcomes, respondents reported using several methods, including progress notes (*n* = 178, 93%), interviews (*n* = 78, 41%), unstandardized assessments (*n* = 71, 37%), standardized assessments (*n* = 24, 13%), and “other” (*n* = 12, 6%). “Other” reported methods included observation (*n* = 4), tracking skills/objectives (*n* = 3), conversations/stories from participants and caregivers (*n* = 3), and end-of-session reports (*n* = 1). Respondents who currently assess A/TR participant outcomes reported measuring outcomes for various reasons, including the following:

- Tracking participant progress (*n* = 183, 96%),
- Program evaluation (*n* = 108, 57%),
- Acquiring funding (*n* = 103, 54%),
- Program support (e.g., reports to board or participants, *n* = 101, 53%),
- Research (*n* = 12, 6%), and
- “Other” (*n* = 12, 6%).

“Other” responses included demonstrating progress to participants, instructors, or the public (*n* = 8), improving the quality of services (*n* = 3), student projects (*n* = 1) and to support future research (*n* = 1).

Among the 71 respondents using unstandardized assessments, respondents most commonly implemented the assessment before and after a *session*, which was defined as a period of consecutive weeks or months during which A/TR lessons are provided (e.g., a 10 weeks session of A/TR lessons; *n* = 42; 59%). The next most common time to implement the unstandardized assessment was after a lesson, defined as a single A/TR lesson (*n* = 23; 32%). Similarly, of the 24 respondents using standardized assessments, they most commonly implemented the assessment before and after a session (*n* = 17; 71%) or after a lesson (*n* = 4; 17%). Respondents reported spending a median of 15 min (IQR = 10–30) implementing unstandardized assessments and a median of 20 min (IQR = 20–30) implementing standardized assessments.

Respondents identified utilizing several different types of unstandardized assessments, including participant and caregiver surveys (*n* = 18, 25%), “in-house” assessments created by A/TR centers (*n* = 9, 13%), modified standardized assessments (*n* = 2, 2.5%), and surveys completed by A/TR instructors (*n* = 1, 1.4%). The most common standardized assessments were Goal Attainment Scaling [GAS; *n* = 9, 38%, (13)] and the Rider Instruction, Development, and Evaluation System [RIDES, *n* = 3, 13%, (14)]; these both represent a standard manner of setting and measuring progress on individual horsemanship goals. Other standardized assessments measure a diverse range of constructs and were only used by 1–2 respondents each:

- Pediatric Evaluation of Disability Inventory-Computer Adaptive Test [PEDI-CAT, *n* = 2, 8%, (15)],
- Strengths and Difficulties questionnaires [*n* = 2, 8%, (16)],
- Subjective Units of Discomfort Scales [SUDS, *n* = 1 (17)],
- Naples Assessment Tool (*n* = 1, 4%),
- Post-traumatic Stress Disorder Checklist [*n* = 1, 4%, (18)],
- Patient Health Questionnaire [PHQ-9, *n* = 1, 4%, (19)],
- Quality of Life Enjoyment and Satisfaction Questionnaire-Short Form [QLES-Q-SF, *n* = 1, 4%, (20)],
- Military to Civilian Questionnaire [M2C-Q, *n* = 1, 4%, (21)],
- Insomnia Severity Index [ISI, *n* = 1, 4%, (22)], and
- Recreation Therapy Assessment (*n* = 1, 4%).

### 3.3 Importance of identifying a standardized outcome assessment

**Figure 1:**
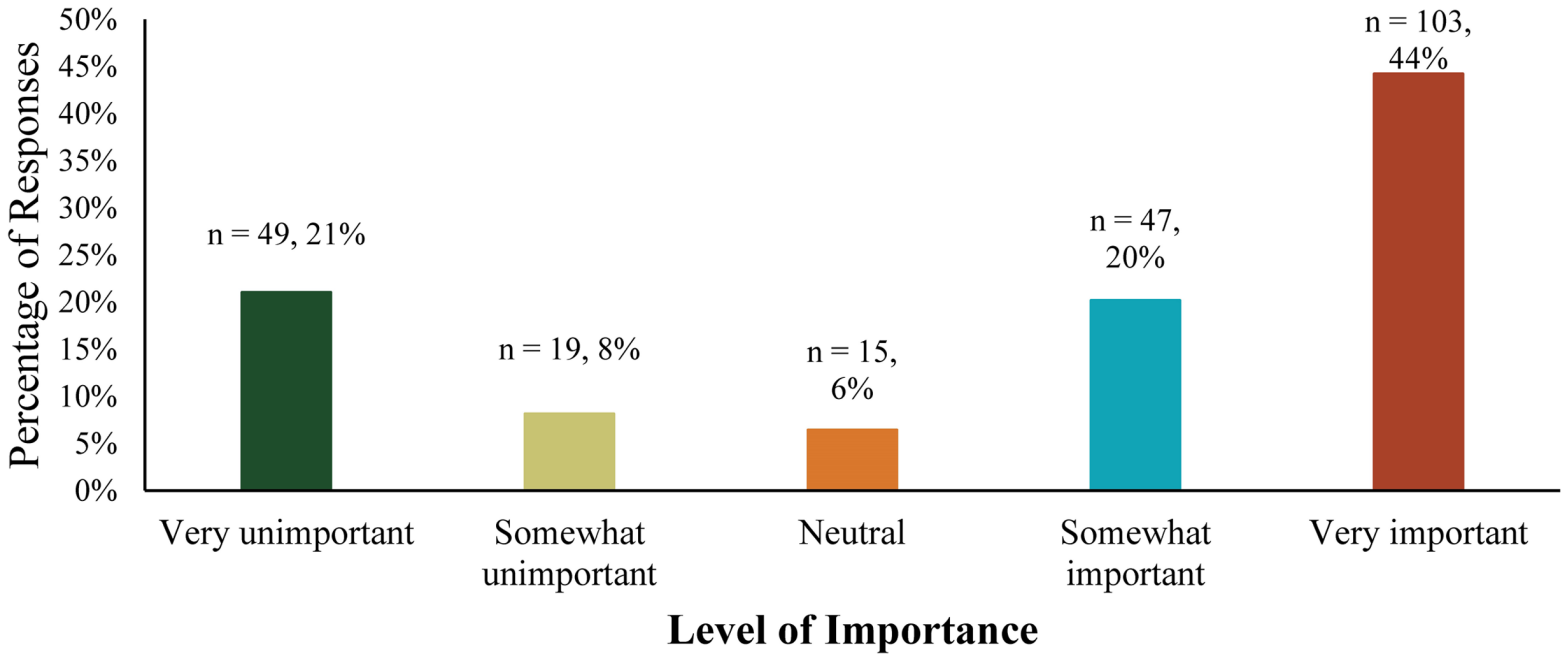
Reported importance of identifying a standardized assessment to measure participant outcomes of A/TR.

Figure 1 illustrates the extent to which CTRIs and PATH, Intl. staff believed it is important to identify a standardized assessment to measure participant outcomes of A/TR. While a notable minority (29%) found it “unimportant” or “very unimportant”, most respondents (64%) reported it was “important” or “very important” to identify standardized assessments to measure participant outcomes of A/TR.

**Figure 2:**
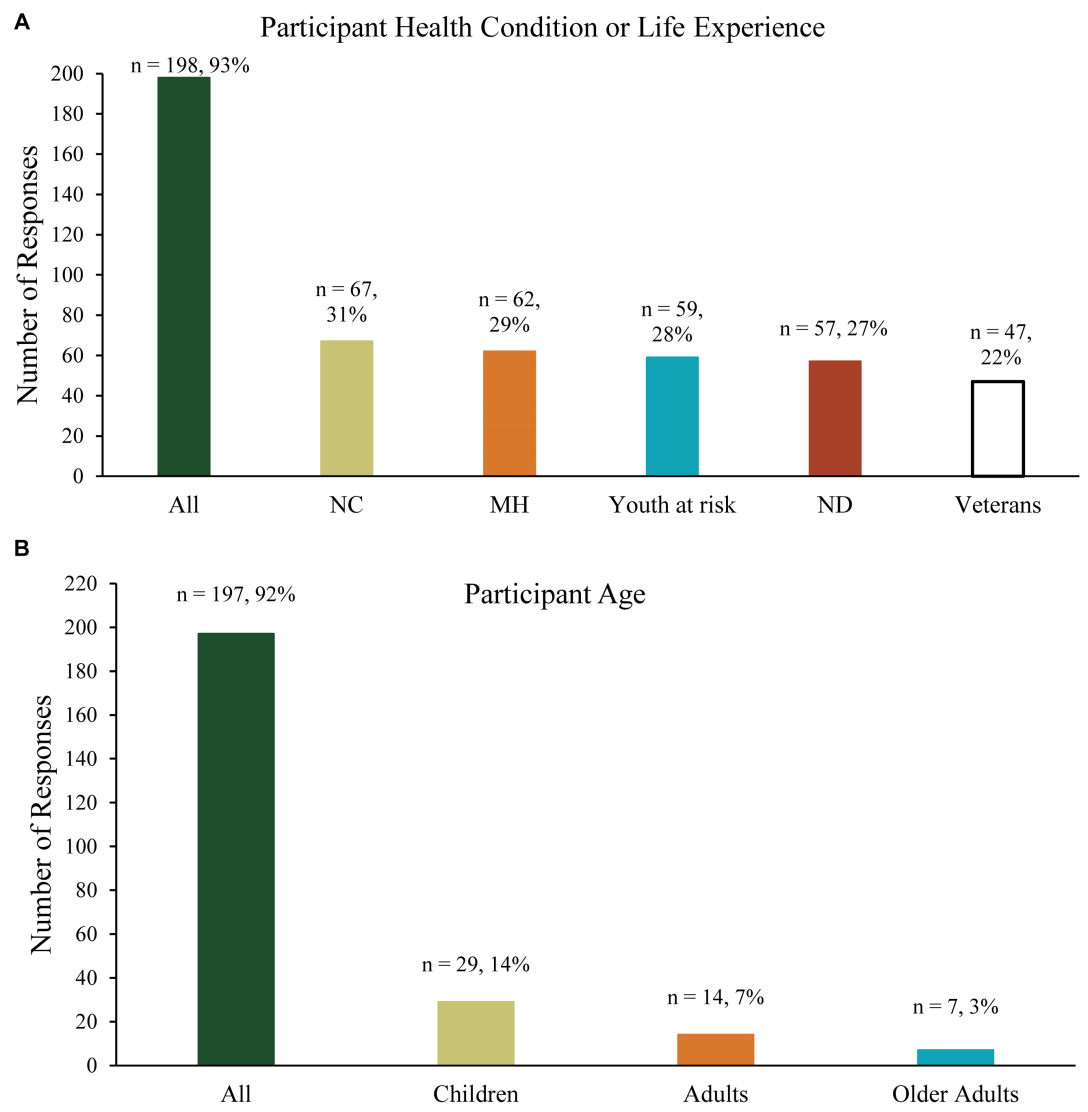
**(A)** Participant age groups identified as important to measure A/TR outcomes in, and **(B)** populations identified as important to measure A/TR outcomes in. NC, neurodevelopmental conditions; MH, mental health conditions; ND, neurological disorders.

Figure 2 illustrates the participant populations for whom respondents believed it would be important to measure outcomes. Over 90% of respondents reported that it would be important to measure A/TR outcomes in all participant age and diagnosis groups.

Table 2 illustrates respondents’ perception of the importance and likelihood to change of several different potential participant A/TR outcomes that could be measured. Twenty-six survey respondents reported “other” outcomes which they believed were important or likely to change. Their responses were coded as: outcome importance depends on individual goals and abilities (*n* = 4, 15%), connection with the horse (*n* = 4, 15%), self-regulation (*n* = 3, 12%), sensory integration (*n* = 2, 8%), relationship building (in a family or between peers; *n* = 2, 8%), academic performance (*n* = 2, 8%), and self-confidence and leadership skills (*n* = 2, 8%).

**Table 2:** Importance and likelihood to improve of participant outcome constructs.

	Importance			Likelihood to improve		
Participant outcome construct	Very unimportant/ unimportant	Neutral	Important/very important	Very unlikely/ unlikely	Neutral	Likely/very likely
Physical	8 (3%)	10 (4%)	215 (93%)	3 (1%)	19 (8%)	202 (89%)
Cognition	6 (3%)	14 (6%)	213 (91%)	0 (0%)	13 (6%)	210 (92%)
Communication	5 (2%)	15 (6%)	213 (91%)	0 (0%)	19 (8%)	206 (91%)
Emotion regulation	9 (4%)	11 (5%)	213 (91%)	2 (1%)	14 (6%)	207 (91%)
Quality of life	5 (2%)	18 (8%)	210 (90%)	3 (1%)	27 (12%)	193 (85%)
Self-efficacy	9 (4%)	15 (6%)	209 (89%)	1 (0.44%)	22 (10%)	202 (89%)
Social	10 (4%)	16 (7%)	207 (89%)	5 (2%)	28 (13%)	190 (84%)
Recreation/leisure	8 (3%)	21 (9%)	204 (87%)	3 (1%)	31 (14%)	190 (84%)
Empathy	7 (3%)	28 (12%)	198 (85%)	1 (0.44%)	5 (2%)	219 (95%)
Horsemanship skills	6 (3%)	32 (14%)	195 (84%)	1 (0.45%)	27 (12%)	195 (85%)
Daily living activities	33 (14%)	74 (32%)	126 (54%)	17 (8%)	122 (54%)	86 (38%)
Community integration	34 (15%)	75 (32%)	124 (53%)	17 (8%)	116 (52%)	92 (41%)
Instrumental activities of daily living	28 (12%)	92 (39%)	113 (49%)	22 (10%)	141 (63%)	60 (26%)

### 3.4 Benefits of and barriers to implementing standardized outcome assessments

Respondents reported several benefits of identifying an assessment to measure A/TR outcomes, including gathering data to demonstrate the value of A/TR to society (*n* = 197, 85%), communicating outcomes to potential A/TR funders (*n* = 183, 79%), communicating outcomes to participants (*n* = 176, 76%), communicating outcomes to future participants (*n* = 172, 74%), guiding how A/TR is delivered or implemented (*n* = 146, 63%), other (*n* = 22, 9%), and none (*n* = 3, 1%). The “Other” responses fell into several categories, including advocating for insurance coverage/funding (*n* = 7, 32%), instructor benefit (*n* = 4, 18%), research (*n* = 3, 14%), communication with other professionals (*n* = 3, 14%), and increasing the credibility of the A/TR profession (*n* = 2, 9%).

Respondents also reported potential obstacles to implementing standardized assessments at their A/TR centers, including: time constraints (*n* = 152, 65%), lack of a system to organize assessments (*n* = 149, 64%), lack of expertise in administering standardized assessments (*n* = 137, 59%), lack of staff to implement assessments (*n* = 118, 51%), assessment cost (*n* = 94, 40%), participant buy-in for assessments (*n* = 59, 25%), lack of knowledge about which assessments to implement (*n* = 48, 12%), other (*n* = 26, 11%), and “none” (*n* = 12, 5%). “Other” responses were coded into the following categories: diversity of PATH, Intl. centers and services provided (*n* = 7, 27%), individual participant variability (e.g., ages, diagnoses, contexts, cultural considerations; *n* = 7, 27%), participant/family burden (*n* = 3, 12%), increased focus on assessment results rather than providing high-quality services (*n* = 2, 8%), and variability inherent to community-based A/TR (e.g., changes in volunteers/horses, participant absences, *n* = 1, 4%).

Given the perceived benefits of and barriers to implementing outcome assessments, respondents were asked to report how likely they would be to use standardized outcome assessments if they were recommended by PATH, Intl. Most respondents reported they were likely (46%, *n* = 108) or very likely (39%, *n* = 90) to implement recommended standardized assessments, while fewer reported they were unlikely (12%, *n* = 28) or very unlikely (3%, *n* = 7).

### 3.5 Usefulness of implementing A/TR outcome assessments

Most survey respondents (*n* = 178, 76%) reported that CTRIs would be the best individuals to report on A/TR participant outcomes, followed by A/TR participant caregivers (*n* = 134, 58%), A/TR participants (*n* = 118, 51%), and “other” (*n* = 31, 13%). Respondents indicated that they would be willing for CTRIs to complete assessments that require a median length of 20 min (IQR = 10–22.5), and that they would feel comfortable asking A/TR participants to complete assessments lasting a median length of 10 min (IQR = 10–20). Additionally, of the 221 total survey respondents, 97 respondents (44%) indicated that they would prefer assessments via a computer/iPad, 94 respondents (43%) identified that they would implement assessments in either computer/iPad or pencil/paper format, and 30 respondents (14%) stated that they would prefer pencil/paper assessments. Finally, most respondents identified that that they would only use assessments that are either free (*n* = 106, 48%) or cost $1–2 per use (*n* = 79, 36%). Fewer respondents stated that they would use assessments costing $3–5 per use (*n* = 33, 15%) or $6–10 per use (*n* = 3, 1.4%).

## 4 Discussion

The current study gathered feedback from CTRIs and PATH, Intl center leadership pertaining to the current use of and attitudes towards standardized assessments to measure participant outcomes of community-based A/TR. As further discussed below, results substantiated there is an existing need across the A/TR industry to identify standardized assessments that can be used for this purpose, and elucidated qualities of standardized assessments that would be *useful* in the community-based A/TR setting.

### 4.1 Substantiating the need for standardized assessments in the A/TR industry

Most respondents reported currently measuring participant outcomes of A/TR participation, primarily through progress notes, interviews, or un-standardized assessments. While these commonly reported methods of recording participant information may be useful to track progress or communicate about participants within centers, they do not easily facilitate communication across centers, between professionals, and to the public. Outward Bound, an organization that provides outdoor education programs, provides an example of how a community-based recreation and learning program can benefit from a standardized assessment to measure participant outcomes. The *Outward Bound Outcomes Survey* “allows all 11 regional Outward Bound schools to collect consistent data on how students are impacted by their Outward Bound experiences in key areas of social–emotional development” (23). Therefore, this comparable program that provides recreational access to both general and vulnerable populations may serve as a model for how the A/TR profession may begin using standardized assessments to measure participant outcomes, facilitating communication between centers and to the public.

While the majority of respondents (64%) reported that it is important to identify standardized assessments to measure outcomes of A/TR participation, a notable minority (29%) of respondents did not think it was important. The rationale for those respondents who reported that it is “not important” to identify a standardized outcome assessment is not clear, as respondents did not have the opportunity to describe why they selected their answers. Other authors have reported that community-based service professionals feel that standardized assessments may not reflect the unique characteristics of their community, programs, and the skills and resources of their participants (24). Furthermore, education on standardized assessments is not included in CTRI training materials (25); therefore, the “unimportant” responses could result from a lack of knowledge about standardized assessments. Despite this disagreement among respondents on the importance of standardized assessments, a large majority of respondents stated they would be either likely or very likely to implement standardized assessments recommended by PATH, Intl. Therefore, results of this survey suggest that if standardized assessments were available and recommended for A/TR professionals, they would be likely to use them. However, the lack of standardized assessment availability is indicated by the fact that only 13% of respondents reported currently using standardized assessments. Therefore, this study substantiated that there is currently an unmet need to identify or develop standardized assessments to measure participant outcomes of community-based A/TR.

#### 4.1.1 Current assessments

The current survey collected information from the few respondents who are using standardized assessments, which may provide helpful insights for identifying ideal assessments to be used in the A/TR context. The most frequently used standardized assessments measure improvements in *horsemanship skills*, the primary focus of A/TR services. The Therapeutic Riding Assessment Impact Network [TRAIN; (26)] designed a TR-specific goal-attainment scaling process that was implemented by 9 respondents; this process involves the development and rating of goals to measure changes in horsemanship skills as a result of participating in A/TR. The next most prevalent standardized assessment, the RIDES tool, was developed by an A/TR center to assess horsemanship skills, develop goals, and track goal progress (14). The RIDES and TRAIN assessment tools could be valuable resources for A/TR professionals interested in measuring improved horsemanship abilities. However, they do not measure the natural benefits of horsemanship in participants’ everyday lives outside of the equine context (e.g., physical outcomes and cognitive outcomes, etc.), which were more often identified by respondents as important constructs to measure (see Table 2).

There was much less consensus among survey respondents regarding standardized assessments currently used to measure the natural health or wellbeing benefits of horsemanship, outside the equine context. This reflects the diverse populations who participate in A/TR and the myriad of benefits which engaging with A/TR provides (2). However, survey results also highlight the difficulty of communicating A/TR outcomes across different centers, and further substantiates the need to identify or develop standardized assessments that can be used across several diverse PATH, Intl centers.

#### 4.1.2 Benefits of and barriers to implementing standardized outcome assessments

The benefits of implementing standardized A/TR assessments reported by survey respondents mirror national trends for program development and evaluation. In recent decades, there has been a national emphasis on evidence-based policies and programs, including community-based programs. Program funders, such as policymakers and funding agency leaders, often require programs to demonstrate their efficacy through research or program evaluation (27). Additionally, for programs to receive national attention and support, they must be evaluated at regular intervals (28). One such method of program evaluation involves using standardized assessments to measure the outcomes which programs claim to address among participants. Standardized assessments are used to measure whether participants are eligible for programs/services, to ensure high quality intervention, and to communicate with internal and external interested parties. This aligns with respondents’ perceived benefits of using standardized assessments in the current survey, which included demonstrating the value of A/TR, communicating with funders and other professionals, advocating for insurance coverage and funding, and bolstering the A/TR profession’s credibility. These responses are similar to benefits of standardized assessments reported in other professions (29–31). Furthermore, consistent use of the same standardized assessments across centers could facilitate consistency in future A/TR research, a stated priority for increasing rigor in human-animal interaction research (7, 32). Overall, the A/TR profession has an opportunity to strengthen its national credibility by implementing standardized assessments to effectively communicate the broad benefits of A/TR participation.

Despite these potential benefits, there are significant barriers to A/TR programs implementing standardized assessments. A/TR programs are not required to implement standardized measures, and training for CTRIs does not include education about how to implement standardized outcome assessments (25). The PATH, Intl course which potential CTRIs are required to take includes a section on participant evaluation and progress notes, but it does not provide education on implementing standardized outcome assessments to track participant progress or measure program effectiveness. Most barriers identified by survey respondents reflect restraints in the A/TR context, including the time, financial, and systemic restraints inherent in A/TR practice. Other professionals have reported similar barriers to those identified by survey respondents, including time, participant burden, and a lack of resources (29–31). These reported barriers should be considered when identifying potentially useful assessments to implement in A/TR settings.

### 4.2 Usefulness of standardized outcome assessments

Given the barriers to using standardized assessments discussed above, it is critical that standardized assessments identified to measure participant outcomes of community-based A/TR are *useful* in the A/TR context. Usefulness is particularly important for A/TR professionals, as they often serve diverse populations with varying needs and abilities and are constrained by the limited time they spend with their participants, limited financial resources, and credentialing restrictions. Over 90% of respondents reported that a useful standardized outcome assessment would be appropriate for use with A/TR participants of all ages and diagnoses/life experiences. Additionally, respondents consistently reported that a wide variety of outcome constructs (i.e., cognitive outcomes, communication outcomes, emotion regulation, and physical outcomes, etc.) are important to measure in A/TR practice. The outcome constructs identified by respondents are consistent with research findings in the A/TR literature; specifically, research has demonstrated that A/TR can improve mental functions (33), social functioning (34), emotional regulation skills (35), communication skills (34), physical function (36, 37), quality of life (38, 39), and community integration (40). Given these findings, a battery of standardized outcome assessments proposed for use in the A/TR setting should measure a wide variety of outcomes in A/TR participants from a variety of age groups, diagnoses, and life experiences. Such a battery of assessments would then likely need to be accompanied by a decision-tool and training to guide A/TR professionals in selecting and implementing the assessment(s) most pertinent to the participants they serve.

Regarding the logistics of implementing standardized assessments, there was not consensus among survey respondents about who should provide information for the assessment (i.e., CTRI-administered assessment vs. participant or caregiver-completed questionnaire), or how the assessment should be delivered (i.e., computer/iPad, paper-pencil, or both). The best standardized assessment format likely depends on individual characteristics of A/TR facilities so it would be beneficial for standardized assessments to be available in both virtual and paper/pencil forms. Most respondents agreed that assessments should not take longer than 10–20 min to administer and should be freely available or low-cost. Therefore, if multiple assessments are identified, they should require no more than 10–20 min total, and the cost for administering assessments should remain minimal.

### 4.3 Limitations

This study includes several limitations. Firstly, respondents were not asked their geographical location or demographic information. These data would have allowed us to understand if trends existed in different areas across the country or based on gender, age, race, or ethnicity. Such demographic data should be included in future studies. Additionally, some sections of the survey did not include a short-answer section, forcing respondents to identify a pre-written response and limiting our capacity to understand the nuances of their answers. Finally, this study is limited by the absence of responses from A/TR participants and their caregivers, whose opinions should be considered in relation to if assessment is important to them, what outcomes should be assessed, and how much time they would be willing to dedicate to participating in an assessment.

### 4.4 Future research directions and conclusions

To our knowledge, this is the first study to gather information from A/TR professionals about the potential usefulness of standardized assessments in community-based A/TR services. Future research should identify or develop standardized assessments that may be useful. To this end, a Delphi study of A/TR experts could aid in developing a prioritized list of potential standardized assessments to measure participant outcomes of A/TR. Once assessments are identified, they should then be piloted in community-based A/TR to understand whether they are feasible in actual A/TR environments. Future directions may also include bolstering community-academic partnerships, to support the implementation of standardized assessments in community-based A/TR. Overall, implementation of standardized assessments in A/TR could enhance the credibility of the profession and provide a means for communicating the vast benefits of community-based A/TR to a variety of audiences.

## Data availability statement

The raw data supporting the conclusions of this article will be made available by the authors, without undue reservation.

## Ethics statement

The studies involving humans were approved by Colorado State U IRB #1, Colorado State University. The studies were conducted in accordance with the local legislation and institutional requirements. The ethics committee/institutional review board waived the requirement of written informed consent for participation from the participants or the participants’ legal guardians/next of kin because the study was designated as minimal risk, and the survey was completely anonymous. Written informed consent would have been the only way to link participant information to the study if it were obtained. Participants indicated consent by reading the consent form and choosing to complete the survey.

## Author contributions

MH: Formal analysis, Writing – original draft. KA: Conceptualization, Data curation, Methodology, Resources, Writing – review & editing. BF: Methodology, Writing – review & editing. RG: Methodology, Writing – review & editing. AS: Supervision, Writing – review & editing. LS: Supervision, Writing – review & editing. BP: Conceptualization, Data curation, Methodology, Supervision, Writing – review & editing.

## References

[1] Wood W Alm K Benjamin J Thomas L Anderson D Pohl L. Optimal terminology for services in the United States that incorporate horses to benefit people: a consensus document. J Altern Complement Med. (2021) 27:88–95. doi: 10.1089/acm.2020.0415 33252244

[2] Professional Association of Therapeutic Horsemanship, International. PATH International Fact Sheet 2020. Available at: https://pathintl.org/wp-content/uploads/2022/03/PATH-facts-2022.pdf (Accessed September 11, 2023).

[3] Bass MM Duchowny CA Llabre MM. The effect of therapeutic horseback riding on social functioning in children with autism. J Autism Dev Disord. (2009) 39:1261–7. doi: 10.1007/s10803-009-0734-3, PMID: 19350376

[4] Farias-Tomaszewski S Jenkins SR Keller J. An evaluation of therapeutic horseback riding programs for adults with physical impairments. Ther Recreat J. (2001) 35:250–7.

[5] Johnson RA Albright DL Marzolf JR Bibbo JL Yaglom HD Crowder SM. Effects of therapeutic horseback riding on post-traumatic stress disorder in military veterans. Mil Med Res. (2018) 5:3. doi: 10.1186/s40779-018-0149-6, PMID: 29502529 PMC5774121

[6] Whalen CN Case-Smith J. Therapeutic effects of horseback riding therapy on gross motor function in children with cerebral palsy: a systematic review. Phys Occup Ther Pediatr. (2012) 32:229–42. doi: 10.3109/01942638.2011.619251, PMID: 22122355

[7] Rodriguez KE Guérin NA Gabriels RL Serpell JA Schreiner PJ O’Haire ME. The state of assessment in human-animal interaction research. Hum Anim Interact Bull. (2018) 6:63–81. doi: 10.1079/hai.2018.0022

[8] Pearson Assessments. Qualifications policy. Available at: https://www.pearsonassessments.com/professional-assessments/ordering/how-to-order/qualifications/qualifications-policy.html (Accessed Mar 24, 2022).

[9] Hunsley J Mash EJ. Evidence-based assessment. Annu Rev Clin Psychol. (2007) 3:29–51. doi: 10.1146/annurev.clinpsy.3.022806.091419 17716047

[10] Harris PA Taylor R Minor BL Elliott V Fernandez M O’Neal L. The REDCap consortium: building an international community of software platform partners. J Biomed Inform. (2019) 95:103208. doi: 10.1016/j.jbi.2019.103208, PMID: 31078660 PMC7254481

[11] Harris PA Taylor R Thielke R Payne J Gonzalez N Conde JG. Research electronic data capture (REDCap)-a metadata-driven methodology and workflow process for providing translational research informatics support. J Biomed Inform. (2009) 42:377–81. doi: 10.1016/j.jbi.2008.08.010, PMID: 18929686 PMC2700030

[12] Hsieh HF Shannon SE. Three approaches to qualitative content analysis. Qual Health Res. (2005) 15:1277–88. doi: 10.1177/1049732305276687 16204405

[13] Kiresuk TJ Sherman RE. Goal attainment scaling: a general method for evaluating comprehensive community mental health programs. Community Ment Health J. (1968) 4:443–53. doi: 10.1007/BF01530764, PMID: 24185570

[14] Saddle Up. RIDES: rider instruction, development and evaluation system. Available at: https://www.saddleupnashville.org/rides-rider-instruction-development-and-evaluation-system/ (Accessed Mar 29, 2023).

[15] Kramer JM Liljenquist K Coster WJ. Validity, reliability, and usability of the pediatric evaluation of disability inventory-computer adaptive test for autism spectrum disorders. Dev Med Child Neurol. (2016) 58:255–61. doi: 10.1111/dmcn.12837, PMID: 26104112 PMC4688240

[16] Goodman R. Psychometric properties of the strengths and difficulties questionnaire. J Am Acad Child Adolesc Psychiatry. (2001) 40:1337–45. doi: 10.1097/00004583-200111000-00015 11699809

[17] Tanner BA. Validity of global physical and emotional SUDS. Appl Psychophysiol Biofeedback. (2012) 37:31–4. doi: 10.1007/s10484-011-9174-x 22038278

[18] Blevins CA Weathers FW Davis MT Witte TK Domino JL. The posttraumatic stress disorder checklist for DSM-5 (PCL-5): development and initial psychometric evaluation. J Trauma Stress. (2015) 28:489–98. doi: 10.1002/jts.22059, PMID: 26606250

[19] Kroenke K Spitzer RL Williams JBW. The PHQ-9: validity of a brief depression severity measure. J Gen Intern Med. (2001) 16:606–13. doi: 10.1046/j.1525-1497.2001.016009606.x, PMID: 11556941 PMC1495268

[20] Stevanovic D. Quality of life enjoyment and satisfaction questionnaire–short form for quality of life assessments in clinical practice: a psychometric study. J Psychiatr Ment Health Nurs. (2011) 18:744–50. doi: 10.1111/j.1365-2850.2011.01735.x, PMID: 21896118

[21] Sayer NA Frazier P Orazem RJ Murdoch M Gravely A Carlson KF. Military to civilian questionnaire: a measure of postdeployment community reintegration difficulty among veterans using Department of Veterans Affairs medical care. J Trauma Stress. (2011) 24:660–70. doi: 10.1002/jts.20706, PMID: 22162082

[22] Morin CM Belleville G Bélanger L Ivers H. The insomnia severity index: psychometric indicators to detect insomnia cases and evaluate treatment response. Sleep. (2011) 34:601. doi: 10.1093/sleep/34.5.601 21532953 PMC3079939

[23] Outward Bound. Welcome to the learning lab. (2023). Available at: https://www.outwardbound.org/programs/outward-bound-professional-learning-lab/ (Accessed Jun 12, 2023).

[24] Judd J James Frankish C Moulton G. Setting standards in the evaluation of community-based health promotion programmes— a unifying approach. Health Promot Int. (2001) 16:367–80. doi: 10.1093/heapro/16.4.367, PMID: 11733455

[25] Professional Association of Therapeutic Horsemanship, International. (2022). PATH Intl. Registered Riding Instructor Certification Booklet. Available at: https://pathintl.org/certification/ctri/ (Accessed Nov 11, 2022).

[26] High Hopes. About – high hopes therapeutic riding. (2023). Available at: https://highhopestr.org/about/ (Accessed Apr 5, 2023).

[27] Mccall RB. Evidence-based programming in the context of practice and policy. Soc Policy Rep. (2009) 23:1–20. doi: 10.1002/j.2379-3988.2009.tb00060.x

[28] Groark CJ McCall RB. Community-based interventions and services. Rutter Child Adolesc Psychiatry. (2009):969–88. doi: 10.1002/9781444300895.ch60

[29] Hatfield DR Ogles BM. The use of outcome measures by psychologists in clinical practice. Prof Psychol Res Pract. (2004) 35:485–91. doi: 10.1037/0735-7028.35.5.485

[30] Jette DU Halbert J Iverson C Miceli E Shah P. Use of standardized outcome measures in physical therapist practice: perceptions and applications. Phys Ther. (2009) 89:125–35. doi: 10.2522/ptj.20080234, PMID: 19074618

[31] Piernik-Yoder B Beck A. The use of standardized assessments in occupational therapy in the United States. Occup Ther Health Care. (2012) 26:97–108. doi: 10.3109/07380577.2012.695103 23899135

[32] Tunis SR Clarke M Gorst SL Gargon E Blazeby JM Altman DG. Improving the relevance and consistency of outcomes in comparative effectiveness research. J Comp Eff Res. (2016) 5:193–205. doi: 10.2217/cer-2015-0007 26930385 PMC4926524

[33] Helmer A Wechsler T Gilboa Y. Equine-assisted services for children with attention-deficit/hyperactivity disorder: a systematic review. J Altern Complement Med. (2021) 27:477–88. doi: 10.1089/acm.2020.0482, PMID: 33835856

[34] Gabriels RL Pan Z Dechant B Agnew JA Brim N Mesibov G. Randomized controlled trial of therapeutic horseback riding in children and adolescents with autism Spectrum disorder. J Am Acad Child Adolesc Psychiatry. (2015) 54:541–9. doi: 10.1016/j.jaac.2015.04.007, PMID: 26088658 PMC4475278

[35] Hoagwood K Vincent A Acri M Morrissey M Seibel L Guo F. Reducing anxiety and stress among youth in a CBT-based equine-assisted adaptive riding program. Animals. (2022) 12:2491. doi: 10.3390/ani12192491 36230232 PMC9558534

[36] Homnick TD Henning KM Swain CV Homnick DN. The effect of therapeutic horseback riding on balance in community-dwelling older adults: a pilot study. J Appl Gerontol. (2015) 34:118–26. doi: 10.1177/0733464812467398, PMID: 25548091

[37] Rigby BR Davis RW Bittner MD Harwell RW Leek EJ Johnson GA. Changes in motor skill proficiency after equine-assisted activities and brain-building tasks in youth with neurodevelopmental disorders. Front Vet Sci. (2020) 7:7. doi: 10.3389/fvets.2020.00022, PMID: 32083104 PMC7004954

[38] Fields B Bruemmer J Gloeckner G Wood W. Influence of an equine-assisted activities program on dementia-specific quality of life. Am J Alzheimers Dis Other Dement. (2018) 33:309–17. doi: 10.1177/1533317518772052 PMC10852437 29742908

[39] White-Lewis S Johnson R Ye S Russell C. An equine-assisted therapy intervention to improve pain, range of motion, and quality of life in adults and older adults with arthritis: a randomized controlled trial. Appl Nurs Res. (2019) 49:5–12. doi: 10.1016/j.apnr.2019.07.002, PMID: 31495419

[40] Lassell R Wood W Schmid AA Cross JE. A comparison of quality of life indicators during two complementary interventions: adaptive gardening and adaptive riding for people with dementia. Complement Ther Med. (2021) 57:102658. doi: 10.1016/j.ctim.2020.102658, PMID: 33429038

